# The low endoribonuclease activity and lack of rNMP preference of human mitochondrial topoisomerase 1 protect against ribonucleotide-dependent deletions

**DOI:** 10.1093/nar/gkaf475

**Published:** 2025-06-06

**Authors:** Cyrielle P J Bader, Erika Miyazaki-Kasho, Josefin M E Forslund, Aiswarya Dash, Malgorzata Wessels, Paulina H Wanrooij

**Affiliations:** Department of Medical Biochemistry and Biophysics, Umeå University, 901 87 Umeå, Sweden; Department of Medical Biochemistry and Biophysics, Umeå University, 901 87 Umeå, Sweden; Department of Medical Biochemistry and Biophysics, Umeå University, 901 87 Umeå, Sweden; Department of Medical Biochemistry and Biophysics, Umeå University, 901 87 Umeå, Sweden; Department of Medical Biochemistry and Biophysics, Umeå University, 901 87 Umeå, Sweden; Department of Medical Biochemistry and Biophysics, Umeå University, 901 87 Umeå, Sweden

## Abstract

The incorporation of ribonucleotides (rNMPs) into the nuclear genome leads to severe genomic instability, including strand breaks and short 2–5 bp deletions at repetitive sequences. Curiously, the detrimental effects of rNMPs are not observed for the human mitochondrial genome (mtDNA) that typically contains several rNMPs per molecule. Given that the nuclear genome instability phenotype is dependent on the activity of the nuclear topoisomerase 1 enzyme (hTOP1), and mammalian mitochondria contain a distinct topoisomerase 1 paralog (hTOP1MT), we hypothesized that the differential effects of rNMPs on the two genomes may reflect divergent properties of the two cellular topoisomerase 1 enzymes. Here, we characterized the endoribonuclease activity of hTOP1MT and found it to be less efficient than that of its nuclear counterpart, a finding that was partly explained by its weaker affinity for its DNA substrate. Moreover, while hTOP1 and yeast TOP1 were able to cleave at an rNMP located even outside of the consensus cleavage site, hTOP1MT showed no such preference for rNMPs. As a consequence, hTOP1MT was inefficient at producing the short rNMP-dependent deletions that are characteristic of TOP1-driven genome instability. These findings help explain the tolerance of rNMPs in the mitochondrial genome.

## Graphical Abstract

**Graphical Abstract:**
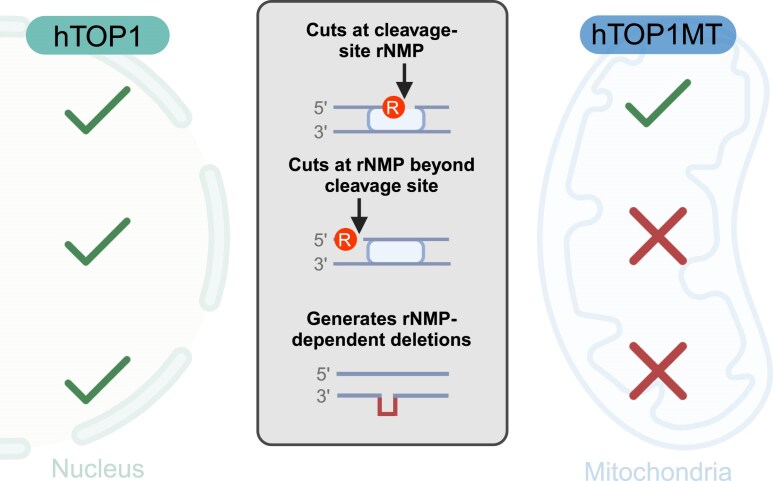

## Introduction

The cellular concentrations of free ribonucleotides (rNTPs) clearly exceed those of deoxyribonucleotides (dNTPs), with a 30–170-fold excess of rNTPs over dNTPs in cycling cells and a 75–1100-fold excess in quiescent cells [1]. Due to this high rNTP/dNTP ratio, single ribonucleotides are occasionally inserted into the nascent DNA strand during DNA replication [2–4]. Although the incorporated ribonucleotides (rNMPs) may have certain positive implications for genome stability such as facilitating some repair processes or relieving torsional stress, the net effects of rNMP incorporation into DNA are negative [5–7]. Even single rNMPs jeopardize genome stability because their reactive 2′-hydroxyl group can induce single-stranded DNA breaks [8]. In addition, incorporated rNMPs alter the local structure and elasticity of the DNA and are thus expected to impact protein–DNA interactions [9–11].

To avoid these harmful consequences, rNMPs incorporated during nuclear DNA replication are efficiently removed by the ribonucleotide excision repair (RER) pathway that is initiated by RNase H2 [12–15]. The importance of rNMP removal is highlighted by the fact that mouse cells deficient in RNase H2 exhibit extensive genome instability in the form of increased DNA breaks, micronuclei, and an activated DNA damage response [12, 13]. In the absence of RER, a lower level of rNMP removal can be mediated by the endoribonuclease activity of topoisomerase 1 (TOP1) [16, 17]. However, TOP1-mediated rNMP repair generates a nick flanked by a 2′,3′-cyclic phosphate that requires further processing prior to religation [18]. At repetitive sequences where strand slippage can occur, TOP1-mediated rNMP repair yields a distinct mutational signature consisting of 2–5 bp deletions [16, 17, 19]. Accordingly, the frequency of these 2–5 bp deletions as well as many of the other genome instability phenotypes of RNase H2-deficient cells is suppressed by the perturbation of TOP1 in both yeast and human cells [17, 20, 21].

RNase H2 and thus RER are absent from the mitochondrial compartment, whereby rNMPs incorporated during the replication of the mitochondrial genome (mtDNA) are not efficiently repaired but instead persist in this small multi-copy genome [22–28]. Based on our knowledge of rNMP biology in the nuclear genome, this absence of RER would be expected to predispose the mtDNA to rNMP-dependent instability [1]. Yet, to our knowledge there have been no reports of short rNMP-dependent deletions in mtDNA. Moreover, our previous work found no beneficial effects of decreasing the mtDNA rNMP load in mice *in vivo*, suggesting that the physiological level of rNMPs in mtDNA is well tolerated [23]. Given that many of the negative effects of rNMPs on the nuclear genome are associated with their removal via the TOP1-mediated repair pathway, we hypothesized that the tolerance of rNMPs in the mitochondrial genome may derive from different propensities of TOP1-dependent ribonucleotide removal in the two cellular compartments.

In addition to the TOP1 found in the nucleus, vertebrates contain a second TOP1 paralog that is restricted to the mitochondrial compartment, TOP1MT [29, 30]. While TOP1MT is not essential, TOP1MT^−/−^ mice exhibit an increase in negatively supercoiled mtDNA and TOP1MT-deficient MEFs manifest with a defect in mitochondrial respiration [31, 32]. Thorough molecular analyses indicate a role for TOP1MT in both mtDNA replication and transcription [30]. Human TOP1MT (hTOP1MT) and the human nuclear TOP1 (hTOP1) share over 70% identity over the core and C-terminal domains that are the ones required for activity [29, 33]. Both hTOP1MT and hTOP1 belong to the Type 1B family of topoisomerases that can relax both negative and positive supercoils. Eukaryotic TOP1 enzymes show strong preference for cleavage following the 3′ thymidine in the consensus sequence motif of 5′-(A/T)(G/C)(A/T)**T**^↓^-3′ [34]. Their reaction mechanism involves the nicking of one strand in a double-stranded DNA (dsDNA) substrate to form a covalent intermediate—termed the TOP1-cleavage complex (TOP1-cc)—between the active site tyrosine of the enzyme and the 3′ thymidine at the nick site [34]. After rotating the substrate to relieve torsional strain, the enzymes catalyze religation of the nick, consequently becoming released from the covalent complex [34]. The reaction can be trapped in the TOP1-cc stage using topoisomerase poisons like camptothecin (CPT) that inhibit the religation step [35].

**Figure 1.**
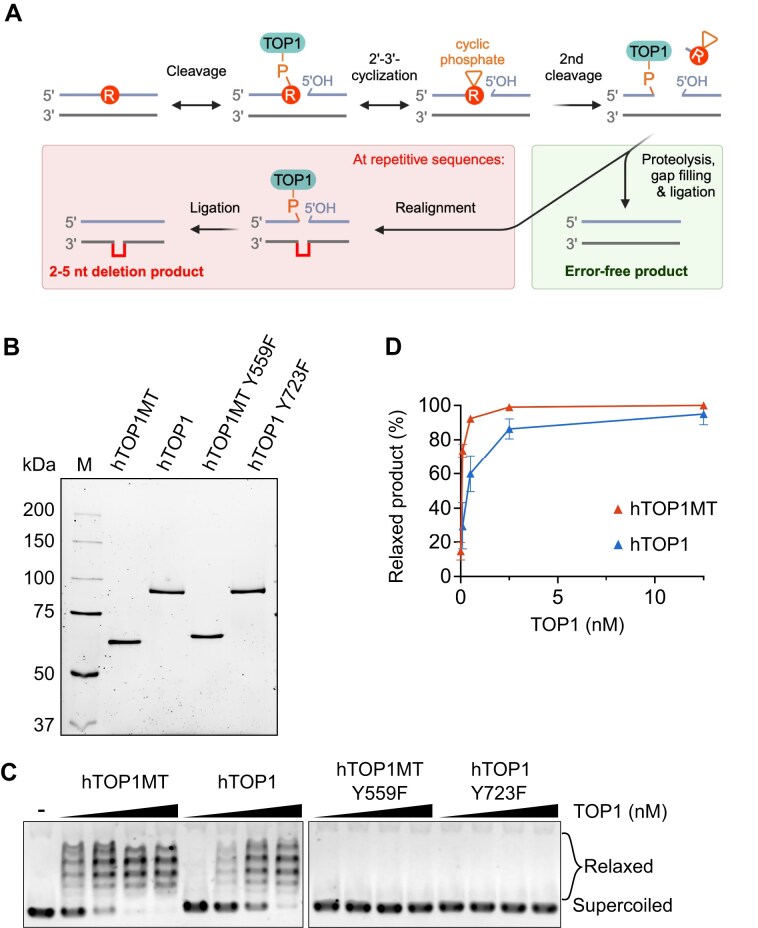
Relaxation of supercoiled DNA by purified recombinant wild-type (WT) hTOP1MT and hTOP1. (**A**) A model of TOP1 action at an incorporated rNMP (circle with "R"). TOP1 cleavage at the rNMP generates a TOP1-cc at the 3′-DNA end, along with a 5′-hydroxyl group. Attack by the 2′-OH of the rNMP on the 3′-phosphotyrosyl bond can prematurely release the TOP1, creating a 2′,3′-cyclic phosphate (open triangle) that cannot undergo ligation without further processing. A second TOP1 cleavage upstream of the cyclic phosphate can release a short DNA fragment containing the modified nucleotide, leaving a gapped intermediate with a covalently associated TOP1 that is too far from the 5′-OH for efficient ligation (top row, far right). An error-free product can be generated via proteolytic removal of the TOP1, followed by gap filling and ligation (bottom right panel). Alternatively, strand realignment at a repetitive sequence can bring TOP1 close enough to the 5′-OH to catalyze ligation of the two DNA ends, leading to deletion of one repeating unit of the repeat sequence in the top strand (bottom left panel; the sequence complementary to the deleted region is shown as a red line). (**B**) SDS–PAGE analysis of the WT and catalytically dead variants of hTOP1MT and hTOP1. (**C**) Relaxation reactions containing 350 ng of supercoiled pUC19 and increasing concentrations (0.1, 0.5, 2.5, and 12.5 nM) of WT (left panel) or catalytically inactive (right panel) hTOP1MT and hTOP1 enzymes. (**D**) Quantification of the relaxation activity of the WT topoisomerases in panel (C). The amount of relaxed product was quantified and expressed in percent of the total signal intensity in the lane. The average of three independent experiments is shown, and the error bars represent the standard error of the mean.

When TOP1 cleavage occurs at an embedded rNMP, the 2′-hydroxyl group of the rNMP can attack the TOP1-cc phospho-tyrosyl bond to generate a 2′,3′-cyclic phosphate and liberate the enzyme prior to religation [16] (Fig. 1A). This results in a nick flanked by a 2′,3′-cyclic phosphate and a 5′-hydroxyl group that cannot be ligated without further processing. Meticulous work on the *Saccharomyces cerevisiae* TOP1 (scTOP1) has revealed that the 2′,3′-cyclic phosphate is a preferred substrate of the enzyme and is rapidly turned over via one of two possible mechanisms: either scTOP1 reverses the cyclization to regenerate the original rNMP-containing substrate or it cleaves two nucleotides upstream of the nick to release a dinucleotide containing the 2′,3′-cyclic phosphate, generating a short gap [19]. Enzymatic removal of the TOP1-cc from this second, upstream cleavage event and filling of the short gap results in an intact, rNMP-free DNA molecule (Fig. 1A, green background). Alternatively, at repetitive sequences that allow strand slippage to occur, the two DNA ends flanking the gap can be brought into close enough proximity for scTOP1 to mediate their ligation (Fig. 1A, red background). Due to the strand realignment, this pathway leads to loss of one of the repeats in the repetitive sequence, explaining the characteristic signature of TOP1-dependent 2–5 nt deletions at repetitive sequences [16, 17, 19].

Like scTOP1, the human nuclear hTOP1 possesses endoribonuclease activity that results in short deletions at repetitive sequences [16, 21]. However, whether the mitochondrial enzyme hTOP1MT contains endoribonuclease activity has not been assessed. In this work, we sought to compare the endoribonuclease activities of the purified human nuclear and mitochondrial TOP1 enzymes and found the mitochondrial enzyme to be less active on rNMPs than its nuclear paralog. This difference in ribonuclease activity was accordingly reflected in the enzymes’ ability to generate short deletions at repetitive sequences. These findings help explain why rNMPs are tolerated in the human mitochondrial genome.

## Materials and methods

### Overexpression and purification of recombinant protein

WT and catalytically inactive variants of hTOP1MT (excluding the mitochondrial targeting sequence), hTOP1, and scTOP1 were cloned into pRS424-GAL-GST where a recognition sequence for the rhinoviral 3C protease separates the GST tag from the N-terminus of the TOP1 gene [36]. Due to restriction enzyme choice during cloning, the hTOP1MT-Y955F variant contains 11 amino acid residues more (DIKLIDTVDLE) from the pRS424-GAL-GST plasmid than the WT enzyme fused to its N-terminus. The presence or absence of these 11 plasmid-backbone-encoded residues had no impact on the activity of the enzyme (data not shown). Recombinant TOP1 variants were expressed in *S. cerevisiae* strain PY116 (*MATa his 3–11,15 leu2-3,112 ura 3–52 trp1-Δ pep4–3 prb1–1122 prc1–407 nuc1::LEU2* [37]); cell lysis and ammonium sulfate precipitation (0.31 g/ml) were essentially as previously described [36], with the following modifications: cells were grown in a LEX-48 bioreactor (Epiphyte 3), and lysed in the 6875 Freezer/Mill (SPEX) using seven cycles with the following settings: *T*_1_ = 2 min, *T*_2_ = 2 min, *T*_3_ = 5 min; impact frequency rate: 12 times/s.

Ammonium sulfate precipitated protein was resuspended in buffer HEP-0 [50 mM HEPES–NaOH, pH 7.4, 10% glycerol, 1 mM dithiothreitol (DTT), 1 mM EDTA, and 0.01% NP-40] and buffer added until the conductivity of the lysate equaled that of HEP-400 (HEP-0 with 400 mM NaCl). The lysate was added to equilibrated glutathione-Sepharose 4B beads (GE Healthcare; 1 ml/100 g cells) and gently rotated for 4 h at 4°C. The beads were collected at 500 × *g* in a swinging-bucket rotor, followed by batch washes (2 × 20 ml of HEP-400). The beads were transferred to a 10-ml column and washed with 40 ml of HEP-400 containing 1 mM pepstatin A, 1 mM AEBSF–HCl, 0.04 mM aprotinin, 0.7 mM E-64, and 1.1 mM leupeptin (Serva), and then with 25 ml HEP-400 containing 5 mM MgCl_2_ and 1 mM ATP. This was followed by a wash with HEP-400 and then HEP-200. Protein was eluted in HEP-150 containing 30 mM reduced glutathione (pH adjusted to 8.1). Fractions that contained TOP1 based on SDS–PAGE (polyacrylamide gel electrophoresis) analysis were pooled and treated with 30 U rhinoviral 3C protease overnight at 4°C.

The following day, the TOP1 protein was loaded onto a heparin column equilibrated in HEP-150 without protease inhibitors. The protein was washed with 10 column volumes of HEP-300 at a flow rate of 1.0 ml/min, and eluted in HEP-750 at a flow rate of 0.5 ml/min. Fractions containing pure protein were collected and dialyzed overnight against HEP-200 in a 10 000 MWCO Slide-A-Lyzer dialysis cassette (Thermo Scientific). Protein concentration was determined by densitometric analysis from SDS–PAGE gels containing a standard curve of bovine serum albumin (BSA; Thermo Scientific).

### Preparation of dsDNA substrates

Oligonucleotides were purchased from Sigma Aldrich, IDT, or Eurofins and are listed in Supplementary Table S1. One nanomole of each oligonucleotide was annealed to an equimolar amount of its complementary strand by denaturing at 95°C for 5 min in TE (50 mM Tris–HCl, pH 8.0, 1 mM EDTA) containing 100 mM NaCl, and allowing the reaction mixture to cool to room temperature. The DNA was separated on a 15% acrylamide gel in 0.5× TBE (15 mM Tris, 44.5 mM boric acid, and 1 mM EDTA), stained with 3× GelRed (Biotium) for 30 min, and visualized by using Chemidoc^™^ (Bio-Rad). The bands corresponding to double-stranded molecules were excised, eluted from crushed gel slices into TE buffer (10 mM Tris–HCl, pH 8.0, 1 mM EDTA), and purified by phenol–chloroform extraction and isopropanol precipitation.

### DNA relaxation assay

The standard cleavage assay mixture contained 11 nM supercoiled pUC19, 10 mM Tris–HCl (pH 8.0), 50 mM KCl, 5 mM MgCl_2_, 0.1 mM EDTA, 5 mM DTT, 15 mg/ml BSA, and the indicated concentration of TOP1 enzymes. Reactions were performed at 30°C for 10 min and terminated by addition of 0.4% SDS and 6× loading buffer (#R0611, Thermo Scientific). The reaction mixture was loaded onto an 0.8% agarose gel in 1× TAE (40 mM Tris, 1 mM EDTA, and 20 mM acetic acid) and separated at 120 V for 2 h. After electrophoresis, DNA bands were stained with 3× GelRed, and visualized on the ChemiDoc imager (Bio-Rad).

### Cleavage assay

The standard 10 μl assay mixture contained 20 mM Tris–HCl (pH 7.8), 5 mM MgCl_2_, 50 mM NaCl, 100 mg/ml BSA, 1 mM DTT, 10 mM DMSO, 50 nM Cy5-labeled dsDNA substrate, and the indicated concentrations of TOP1 in the presence or absence of 10 μM CPT. Reactions were carried out at 30°C for 60 min and terminated by addition of 5 μl of 5× Stop buffer (10 mM EDTA, 0.05% SDS) and 10 μl of formamide. Samples were resolved on a 17% PAGE gel containing 7 M urea in 1× TBE at 60 W for 2.5 h. Fluorescence signal was detected on a Typhoon Biomolecular imager (Amersham) and quantified using ImageJ software (Java).

### Steady-state kinetic analysis of rNMP cleavage

The enzyme kinetics of rNMP cleavage by hTOP1 and hTOP1MT were measured using a gel-based assay. Twenty nanomolar hTOP1 or hTOP1MT was incubated with 2.5–60 nM Cy5-labeled substrate B in the standard cleavage assay reaction buffer (20 mM Tris–HCl, pH 7.8, 5 mM MgCl_2_, 50 mM NaCl, 100 mg/ml BSA, 1 mM DTT) in a total volume of 10 μl. Reactions were terminated at different time points (0.5–8 min for hTOP1, 15–60 min for hTOP1MT) by the addition of 10 μl Stop buffer (final concentration 47.5% formamide, 0.5% SDS, and 25 mM EDTA), boiled for 5 min, and separated on a 17% polyacrylamide gel with 7 M urea for 2 h 20 min. The gel was scanned using Amersham Typhoon imager (Cytiva) and quantitative analysis of three independent experiments was carried out using ImageQuantTL software (Cytiva). The intensity of the 18-nt cleavage product was quantified and converted to concentration (pM) using a standard curve run on the same gel. Product concentration (*y*) was plotted against time (*x*, in seconds) at each substrate concentration. Initial velocities, *V*_0_, at each substrate concentration were determined from the slope and plotted against the substrate concentration. The steady-state kinetic values (*K*_m_ and *V*_max_) were determined using nonlinear regression analysis in GraphPad Prism 10 software, and *k*_cat_ values were calculated by dividing *V*_max_ with the concentration of topoisomerase used.

### Fluorescence anisotropy

Fluorescence anisotropy reactions containing 0.5 nM double-stranded oligonucleotides with a 5′-FAM-labeled top strand and 0–60 nM TOP1 in binding buffer (20 mM Tris, pH 8, 66 mM NaCl, 1 mM DTT, 2% glycerol) were pipetted in triplicate onto black shallow 384-well microplates (OptiPlate-F, PerkinElmer) and incubated in the dark for 10 min at 37°C. Fluorescence intensities were measured from above on a CLARIOstar Plus plate reader (BMG Labtech) with the excitation and emission wavelengths of 480 and 520 nm, respectively. Fluorescence anisotropy in millianisotropy units (mA) was calculated using MARS Data Analysis Software (BMG Labtech) according to equation (1): fluorescence anisotropy = ($F_\parallel$ − $ F_\bot$)/ ($F_\parallel$ + 2 × $F_\bot$) × 1000, where $F_\parallel$ and $ F_\bot$ are the parallel and perpendicular emission intensity measurements corrected for background (buffer). The grating factor *G* was calculated using equation (2): *G* = $F_\parallel$/$ F_\bot$ and was equal to 1. The dissociation constant (*K*_d_) was determined by fitting the data to a quadratic equation by nonlinear regression analysis in GraphPad Prism software (GraphPad Software, Inc., USA) using equation (2):


\begin{eqnarray*}
{{Y}} = {{B}}_0 + \left( {{{B_{\rm max}}} - {{B}}_0} \right) \times \frac{{\sqrt {{\mathrm{\ }}{{{\left( {{{D}} + {{X}} + {{K_{\rm d}}}} \right)}}^2} - {\mathrm{\ }}\left( {4 \times {{D}} \times {{X}}} \right)} }}{{2 \times {{D}}}} ,
\end{eqnarray*}

where *Y* is the anisotropy value at protein concentration *X*, *X* is the concentration of TOP1 in nM, *B*_0_ and *B*_max_ are specific anisotropy values associated with free DNA and the DNA–TOP1 complex, respectively, and *D* is the concentration of DNA in nM. Reactions with different substrate concentrations yielded *K*_d_ values that did not significantly differ from those in Supplementary Table S2 with 0.5 nM substrate, confirming the *K*_d_ values were not artifacts of analysis in the intermediate regime [38].

## Results

### hTOP1MT exhibits endoribonuclease activity

To compare the properties of hTOP1MT and hTOP1, we expressed and purified the recombinant WT proteins along with their catalytically inactive variants where the catalytic tyrosine was mutated to a phenylalanine (Fig. 1B). Both WT enzymes showed robust activity on a negatively supercoiled plasmid, with hTOP1MT catalyzing full relaxation of the substrate at an ∼5-fold lower enzyme concentration than hTOP1 (Fig. 1C and D; 14% supercoiled substrate remaining with 2.5 nM hTOP1 versus 8% with 0.5 nM hTOP1MT). As expected, the catalytically inactive variants were unable to relax the substrate.

**Figure 2.**
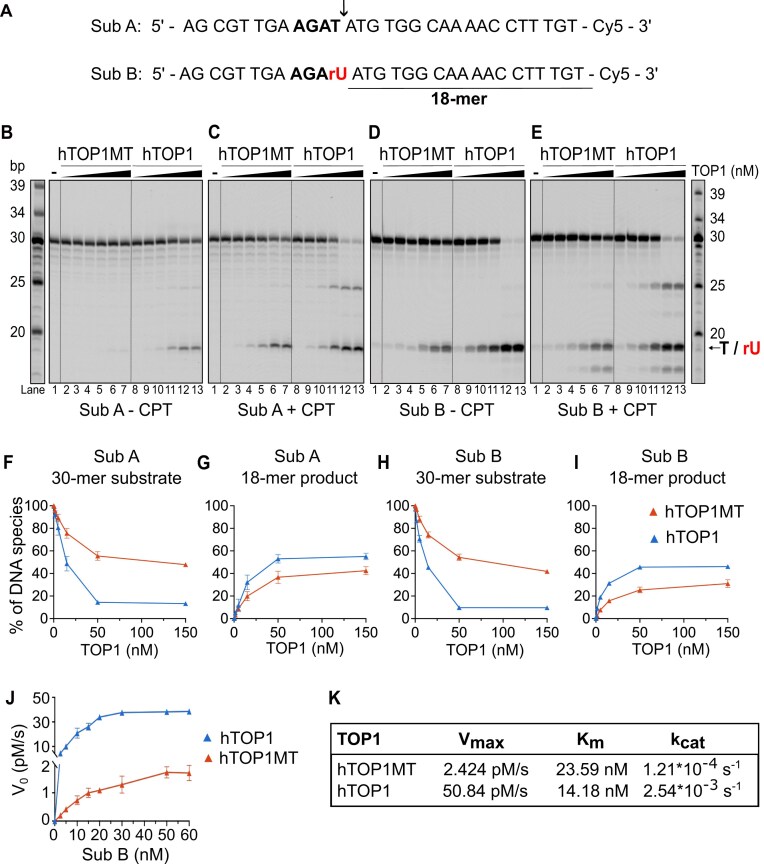
hTOP1MT exhibits endoribonuclease activity. (**A**) The top strand of the dsDNA substrates containing the preferred cleavage motif (bold) found at the (AT)_2_ hotspot of the *S. cerevisiae* CAN1 locus. The consensus cleavage site is marked by a black arrow and the resulting 18-nt product is underlined. The top strand was labeled with Cy5 at the 3′-end. Substrate B contains an rUMP (red) at the cleavage site. Representative TOP1 cleavage assays containing 50 nM substrate A and increasing concentrations (0.15, 0.5, 5, 15, 50, and 150 nM) of WT hTOP1MT and hTOP1 enzymes in the absence (**B**) and presence (**C**) of 10 μM CPT, an inhibitor of the religation step. Cleavage assays on substrate B with an rUMP at the cleavage site in the absence (**D**) and presence (**E**) of CPT; reactions conditions are as in panels (B) and (C). Quantification of the 30-mer substrate (**F**, **H**) and the 18-mer cleavage product (**G**, **I**) from assays on substrate A (F, G) and substrate B (H, I) in the presence of CPT. The intensity of the indicated band is expressed in percent of the total signal intensity in the lane. Only these reactions in the presence of CPT allow comparison of TOP1 activity at the cleavage site containing a dT versus dU; however, the quantification of the no-CPT reactions is shown in Supplementary Fig. S1A and B for reference. The average of the three independent experiments is shown, and the error bars represent the standard error of the mean. (**J**, **K**) Steady-state kinetic analysis of hTOP1MT (red) and hTOP1 (blue) activities on the rNMP-containing substrate B was carried out using the gel-based cleavage assay similar to panel (D). Please note the split *y*-axis. Time course experiments were performed by incubating various concentrations of substrate B (2.5–60 nM) with 20 nM of hTOP1MT or hTOP1. After separation of the reactions on gel, the 18-nt cleavage product was quantified. Initial velocities (*V*_0_) were calculated from three independent replicates, plotted against substrate concentration, and nonlinear regression analysis was used to determine the *V*_max_, *K*_m_, and *k*_cat_ values presented in panel (K).

We next analyzed the ability of the TOP1 enzymes to cleave a linear double-stranded 30-bp substrate containing the perfect consensus cleavage motif 5′-AGA**T**^↓^-3′ from the (AT)_2_ hotspot of the *S. cerevisiae* CAN1 gene that exhibits a strong TOP1-dependent deletion signature [17, 39]. Figure 2A shows the top strand of the double-stranded substrate; the bottom strand is omitted for clarity. The experiments were carried out on a pair of substrates where the top strand consisted of all deoxyribonucleotides (substrate A) or where the deoxy-T at the cleavage site was replaced with a ribo-U (substrate B; rU in red in Fig. 2A). Substrates were fluorescently labeled on the 3′-end of the top strand, whereby cleavage at the preferred cleavage site should yield an 18-nucleotide product that can be readily detected if the religation step of the reaction is prevented either by addition of CPT or through the premature liberation of the enzyme upon 2′–3′-cyclization when the cleavage occurs at an rNMP (Fig. 1A).

Analysis of reactions carried out on the all-DNA substrate A in the presence of CPT revealed that both hTOP1MT and hTOP1 primarily produced the expected 18-nt cleavage product, with some formation of a minor product derived from cleavage at an upstream imperfect cleavage motif (Fig. 2C). In the absence of CPT, product was only detected in hTOP1 reactions with high concentrations of enzyme (Fig. 2B). On substrate B containing an rUMP at the cleavage site, frequent cleavage events at the ribonucleotide were readily detected with both TOP1 enzymes even in the absence of CPT, confirming they occurred at the rUMP (Fig. 2D). Addition of the inhibitor revealed the presence of additional cleavage sites beyond the consensus cleavage site, including one which was located a few nucleotides downstream of the rUMP (Fig. 2, compare panels D and E). This downstream cleavage event was only observed on substrate B and therefore likely represents the reported preference of TOP1 to cleave 2–6 nt from a nick [40], in this case near the persistent TOP1-cc formed by an initial cleavage at the rUMP.

The results of Fig. 2D demonstrate that like its nuclear homolog, the human mitochondrial topoisomerase exhibits endoribonuclease activity. However, cleavage by hTOP1MT appeared to be less efficient than that by nuclear hTOP1 on both the substrate with and without an rNMP at the cleavage site, generating less 18-mer product and thus consuming less of the full-length DNA substrate (Fig. 2F–I; see Supplementary Fig. S1A and B for reactions without CPT). To quantitatively compare the endoribonuclease activities of the two topoisomerases, steady-state kinetic experiments were carried out on substrate B. The results of the kinetic analysis demonstrated that the *V*_max_ of the reaction was 21-fold lower for hTOP1MT compared to hTOP1, while the *K*_m_ values only differed 1.6-fold (Fig. 2J and K). These findings highlight the considerable difference in endoribonuclease activity between the two enzymes, with hTOP1MT cleaving significantly slower at the cleavage-site rNMP than hTOP1.

### hTOP1MT and hTOP1 differ in their propensity to act at an upstream rNMP

**Figure 3.**
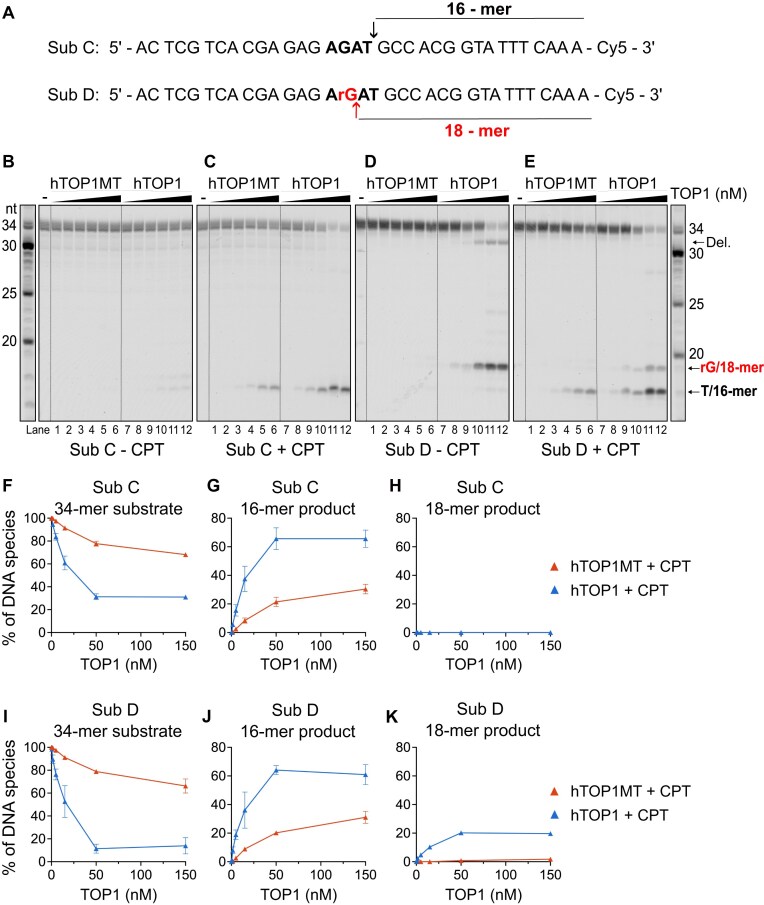
hTOP1MT and hTOP1 differ in their propensity to cleave at an rNMP beyond the preferred cleavage site. (**A**) The top strand of the dsDNA substrates containing the preferred cleavage motif (bold) modified from the (TC)_3_ hotspot of the *S. cerevisiae* CAN1 locus. The top strand was labeled with Cy5 at the 3′-end. The cleavage site is marked by a black arrow and the resulting 16-nt product is indicated. Substrate D contains an rGMP (red) 2 nt upstream of the consensus cleavage site; cleavage at the rGMP generates an 18-nt product (red arrow). Representative TOP1 cleavage assays containing 50 nM substrate C and increasing concentrations (0.15, 0.5, 5, 15, 50, and 150 nM) of WT hTOP1MT and hTOP1 enzymes in the absence (**B**) and presence (**C**) of 10 μM CPT. Cleavage assays in the absence (**D**) and presence (**E**) of CPT were carried out as in panels (B) and (C) but on substrate D with an rGMP upstream of the cleavage site. Del. denotes a deletion product that is produced on substrate D. Quantification of the substrate (**F**, **I**), and the 16-mer (**G**, **J**) and 18-mer (**H**, **K**) cleavage products from assays on substrate C (F–H) and substrate D (I–K) in the presence of CPT. The intensity of the indicated band is presented in percent of the total signal intensity in the lane; the quantification of products in the absence of CPT is shown in Supplementary Fig. S1C–F. The average of three independent experiments is shown, and the error bars represent the standard error of the mean.

Next, we analyzed the preference of the TOP1 enzymes for cleavage at an rNMP over a dNMP. To this end, we used a 34-mer dsDNA substrate derived from another CAN1 deletion hotspot, named the (TC)_3_ after the reverse complement sequence, that has been thoroughly studied in the context of TOP1-mediated rNMP repair [17, 19]. The activity of the two TOP1 proteins was compared on the all-DNA substrate (substrate C) and on substrate D that contained a single rNMP located 2 nt upstream of the preferred cleavage site (Fig. 3A). On this pair of substrates, cleavage at the perfect consensus cleavage site should generate a 16-mer product, while cleavage at the upstream rGMP should yield an 18-mer. The action of both TOP1 enzymes on the all-DNA substrate C yielded only the 16-mer product, indicating that this substrate contained only the cleavage site corresponding to the consensus cleavage motif (Fig. 3B, C, and F–H). However, a longer 18-nt product formed through cleavage at the upstream rGMP was observed in reactions containing the ribonucleotide-containing substrate D both in the absence and in the presence of CPT (Fig. 3D, E, and I–K, and Supplementary Fig. S1C–F). Interestingly, the 18-mer product was only generated by hTOP1 and not by hTOP1MT (Fig. 3K). These results indicate that an embedded rNMP is able to attract hTOP1 to cleave beyond its consensus cleavage site, while hTOP1MT acts only at the consensus site. As seen by the appearance of a 32-mer deletion product in reactions with hTOP1 in Fig. 3D, this behavior has implications for TOP1-dependent deletion formation that will be more closely addressed in Fig. 5.

### hTOP1MT exhibits lower DNA binding affinity than its nuclear paralog

**Figure 4.**
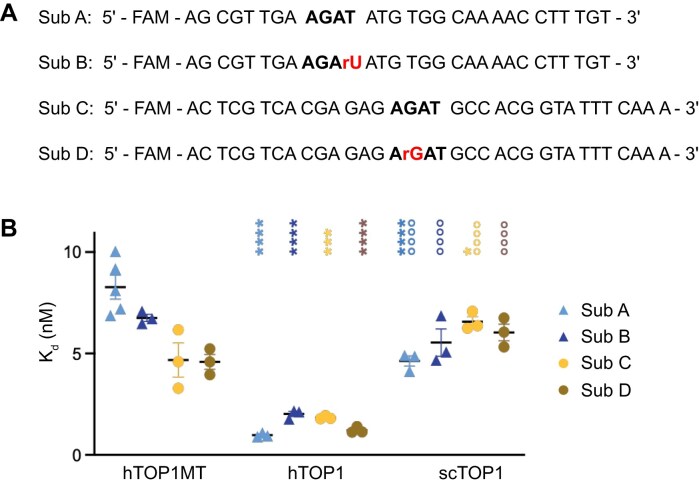
hTOP1MT and scTOP1 bind their DNA substrates with lower affinity than hTOP1. (**A**) The sequences of the 5′-FAM-labeled top strands of the dsDNA substrates used for the fluorescence anisotropy measurements in panel (B). (**B**) The *K*_d_ values of hTOP1MT, hTOP1, and scTOP1 for each of the four substrates A–D were determined based on the anisotropy data in Supplementary Fig. S2 using the quadratic equation. The mean of three to five independent experiments is shown, and error bars represent the standard error of the mean. Topoisomerases and substrates were compared using two-way ANOVA. Asterisks indicate the *P*-value of the comparison with hTOP1MT, and empty circles the one from comparison with hTOP1: *P < .05, ***P < .001, and ****P < .0001. The average *K*_d_ and the *P*-values are presented in Supplementary Table S2.

Prompted by the observed differential cleavage activity of the two human TOP1 paralogs, we next investigated the DNA binding affinity of the TOP1 enzymes using fluorescence anisotropy. In addition to the two human TOP1 proteins, these experiments were expanded to include their *S. cerevisiae* homolog (scTOP1) that is dually localized to the both nuclear and mitochondrial compartments [41] (see Supplementary Fig. S1G–I for SDS–PAGE analysis and DNA relaxation activity of scTOP1). To avoid confounding effects due to differences in enzyme activity and thus the efficiency of covalent complex formation during the enzymes’ reaction cycle, we limited our analysis to noncovalent binding by using the catalytically inactive TOP1 variants, a strategy successfully employed in studies of, for example, the related vaccinia virus TOP1 [42]. Binding was investigated on the two dsDNA substrate pairs used in Figs 2 and 3: substrate A versus substrate B where the rNMP is located at the cleavage site (AGAT^↓^ versus AGArU^↓^), and substrate C versus substrate D with the rNMP located upstream of the cleavage site (AGAT^↓^ versus ArGAT^↓^) (Fig. 4A). The anisotropy values for each TOP1 complexed with each of the four substrates were used to determine their respective *K*_d_ values (Fig. 4B; see Supplementary Fig. S2 for anisotropy data and Supplementary Table S2 for exact *K*_d_ values).

A comparison of the *K*_d_ values demonstrates that hTOP1MT exhibited significantly lower affinity for all four substrates than hTOP1 did, with 3–9-fold higher dissociation constants than hTOP1 (Fig. 4B and Supplementary Table S2). The observed lower affinity of hTOP1MT to DNA is well in line with earlier reports based on the chromatin retainment of hybrid TOP1 enzymes *in cellulo* [43]. This lower DNA binding affinity of hTOP1MT likely contributes to its lower cleavage efficiency on linear DNA substrates with or without an incorporated rNMP (Figs 2G and I, and 3G–K). Also the DNA binding affinity of the yeast scTOP1 enzyme was lower than that of hTOP1 across all four substrates (Fig. 4B and Supplementary Table S2). Finally, the presence of a single rNMP either at the cleavage site (substrate B) or upstream of the cleavage site (substrate D) had no significant impact on substrate binding affinity by any of the topoisomerases when compared to the respective control substrates (substrates A and C, respectively) (Fig. 4B).

In conclusion, the binding behavior of hTOP1MT—as well as that of the yeast enzyme scTOP1—differs from that of the human nuclear homolog hTOP1, which exhibits significantly higher affinity for the tested DNA substrates, regardless of the presence of a single rNMP.

### In contrast to hTOP1 and scTOP1, hTOP1MT action rarely leads to rNMP-dependent deletions

**Figure 5.**
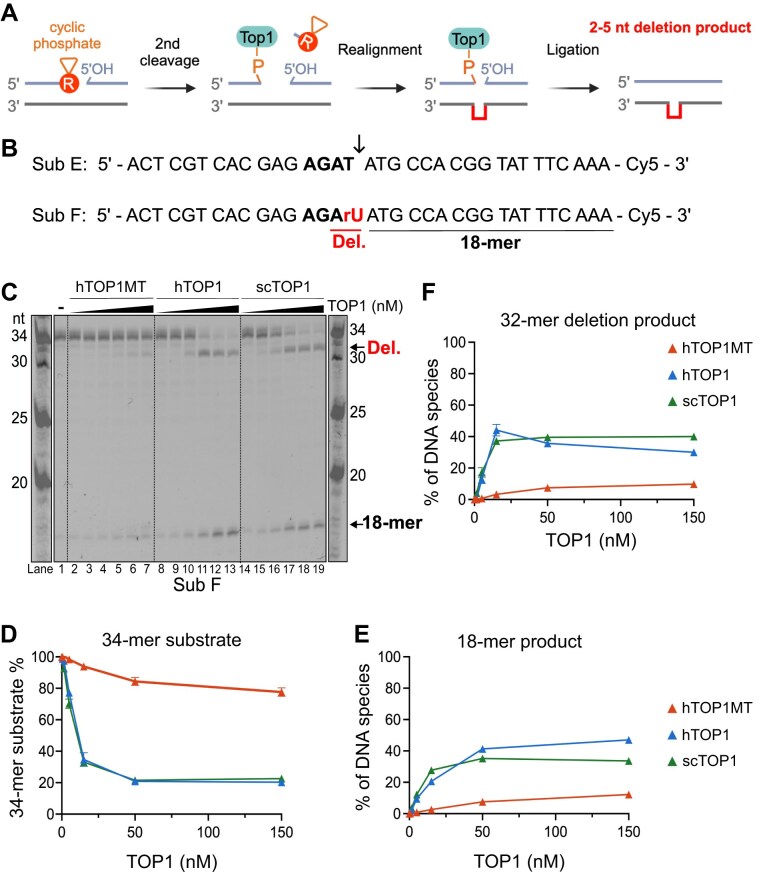
hTOP1MT-dependent deletion formation is a rare event. (**A**) Schematic representation of deletion formation on a dsDNA substrate containing an rNMP at a repetitive sequence in the top strand. The TOP1-cc at the rNMP is dissolved following the formation of a 2′,3′-cyclic phosphate (not shown). A second TOP1 cleavage a few nt upstream releases the fragment with the cyclic phosphate. Realignment of the two strands relative to each other extrudes one repeating unit in the repeat sequence (red line), bringing the 3′-phosphate-TOP1 close enough to the 5′-hydroxyl end for TOP1 to finish its reaction cycle through ligation of the DNA ends. This results in deletion of one repeat unit in the top strand. (**B**) The top strand of the dsDNA substrates E and F containing the preferred cleavage motif (bold) modified from the (TC)_3_ hotspot of the *S. cerevisiae* CAN1 locus. The top strand was labeled with Cy5 at the 3′-end. The cleavage site is marked by a black arrow and the resulting 18-nt product is indicated. Substrate F contains an rUMP (red) at the consensus cleavage site, cleavage at which can result in a 2 nt deletion (red underline). (**C**) Representative TOP1 cleavage assay containing 50 nM substrate F and increasing concentrations (0.15, 0.5, 5, 15, 50, and 150 nM) of WT hTOP1MT, hTOP1, and scTOP1 enzymes in the absence of CPT. Quantification of the 34-mer substrate (**D**), the 18-mer cleavage product (**E**), and the 32-mer deletion product (**F**) in the reactions in panel (C). The intensity of the indicated bands was quantified and expressed in percent of the total signal intensity in the lane. The average of the three independent experiments is shown, and the error bars represent the standard error of the mean.

As mentioned above, the action of hTOP1 and scTOP1 at rNMPs has been shown to promote the generation of 2–5 nt deletions when the rNMP is present at a repetitive sequence that allows strand slippage to occur [16, 17, 19]. Briefly, deletions arise through a mechanism that involves a second TOP1 cleavage event a few nt upstream of the 2′,3′-cyclic phosphate, followed by strand slippage that extrudes one repeating unit of the repeat sequence. This strand realignment brings the TOP1-bound 3′-phosphate end of the DNA close enough to the 5′-hydroxyl end for the enzyme to catalyze ligation of the two ends (Fig. 5A). As a result, one repeating unit of the repeat sequence is deleted from the top strand, and replication of this strand will yield a dsDNA molecule lacking one repeat unit from both strands.

Our results so far indicate that compared to hTOP1, hTOP1MT shows weaker affinity for its DNA substrates, and incises somewhat less efficiently at a cleavage-site rNMP (Figs 2I and 4B). We next asked how efficiently hTOP1MT mediates rNMP-dependent deletion formation at repetitive sequences. For this, the sequence of the CAN1 (TC)_3_ deletion hotspot was modified by insertion of an AT dinucleotide immediately 3′ of the cleavage site, an alteration that is known to increase the efficiency of deletion formation [19]. The resulting substrates E and F contained the consensus T or an rU, respectively, at the cleavage site (Fig. 5B).

The action of hTOP1MT, hTOP1, and scTOP1 on the rUMP-containing substrate F yielded the expected 18-nt cleavage product as well as a 32-mer deletion product (Fig. 5C–F). However, in line with the relatively inefficient cleavage at the rUMP observed with hTOP1MT (Fig. 5E), the yield of deletion product generated by hTOP1MT was significantly lower than with hTOP1 or scTOP1: only 9.7% of the substrate was converted to the 32-mer deletion product by hTOP1MT, compared to 30% and 40% for hTOP1 and scTOP1, respectively (Fig. 5F). As expected for an rNMP-specific deletion mechanism, no deletion formation was observed on the all-DNA substrate E (Supplementary Fig. S3A). Instead, TOP1 action on substrate E yielded only the 18-mer product that corresponds to cleavage at the consensus cleavage site, and this product was only observed in the presence of CPT (Supplementary Fig. S3A and B). Of note is that in the presence of CPT, hTOP1MT action on the all-DNA substrate E generated more of the 18-mer cleavage product than did hTOP1 (Supplementary Fig. S3B). Moreover, the presence of CPT did not affect hTOP1MT cleavage efficiency on the rNMP-containing substrate F (compare Supplementary Fig. S3C and D to Fig. 5E). Together, these results indicate that while hTOP1MT efficiently cleaves at the cleavage motif in the absence of an rNMP, the presence of a ribonucleotide at the cleavage site lowers its cleavage efficiency, a phenomenon that does not occur with hTOP1 or scTOP1. Consequently, hTOP1MT-mediated deletion formation is a rare event.

## Discussion

Previous work in the field of mtDNA maintenance has uncovered a differential impact of the physiological level of rNMPs on mtDNA stability in yeast and mammalian cells—while a decrease in rNMP frequency had a positive impact on mtDNA stability in *S. cerevisiae*, it had no observable effect on the integrity of mouse mtDNA [22, 23]. The different properties of the type 1B topoisomerases illuminated in the current study offer at least one contributing mechanism to explain the observed discrepancy between organisms. Compared to its nuclear counterpart, the human mitochondrial topoisomerase 1 hTOP1MT shows ∼3-fold lower DNA binding affinity for an rNMP-containing substrate, which is likely a contributing factor to the 1.3- to 4-fold lower cleavage efficiency observed at a cleavage site rUMP on substrates B and F, respectively (Figs 2, 4, and 5). In fact, under steady-state kinetic conditions, the maximum velocity of hTOP1MT cleavage on substrate B was 21-fold lower than that of hTOP1 (Fig. 2). As a consequence of its lower endoribonuclease activity, hTOP1MT action resulted in a clearly lower efficiency of deletion formation: at 15 nM TOP1 concentration, when deletion formation was still largely dependent on enzyme concentration, hTOP1MT generated 13-fold less deletion product than hTOP1 (Fig. 5F). Importantly, the lower frequency of deletion formation does not reflect a lower overall activity of hTOP1MT that was able to relax a supercoiled substrate at over five-fold lower enzyme concentration than hTOP1. Our findings of comparatively higher activity of hTOP1MT on supercoiled substrates align well with previous elegant single-molecule analyses demonstrating the higher torque-dependence of hTOP1MT relaxation activity when compared to hTOP1 [44]. Moreover, we speculate that the relatively higher activity of hTOP1MT on supercoiled than on linear substrates may reflect tighter binding to the former, as has been demonstrated for hTOP1 [45]. The binding behavior of the two human TOP1 enzymes on different conformations of substrate should thus be further investigated in the future.

Most strikingly, while hTOP1 can shift to cleave at an rNMP located outside of the consensus cleavage site, the same behavior is not observed for hTOP1MT (Fig. 3). Like hTOP1, also scTOP1 showed efficient cleavage at the rG upstream of the cleavage site on substrate D (Supplementary Fig. S3E–G). This differing feature between hTOP1MT and the other two TOP1 proteins is expected to have a major impact *in cellulo*, where the substrate of hTOP1MT is an mtDNA molecule with a random distribution of incorporated rNMPs. Mammalian mtDNA contains ∼6–31 incorporated rNMPs per strand, which corresponds to an average frequency of one rNMP per 530–2700 nt [23, 24, 46]. Given this low rNMP frequency, an incorporated rNMP is very unlikely to be located precisely at a TOP1 consensus cleavage site, and there will therefore be very few opportunities for hTOP1MT to act at an rNMP. The likelihood is decreased further by the fact that rUMP, which should be the rNMP incorporated at the consensus cleavage site 5′-AGAT^↓^-3′, is the least frequent rNMP found in mammalian mtDNA, in both mouse and human cells [23, 24]. However, based on the findings in Fig. 3 and Supplementary Fig. S3, the properties of hTOP1 and scTOP1 would allow them to act even at an rNMP outside the consensus cleavage site and thus potentially generate deletions and/or nicked repair intermediates at far more positions than hTOP1MT. Taken together, the properties of hTOP1MT uncovered here help explain why rNMPs have less of a negative impact on mtDNA in mammalian compared to yeast cells.

A comparison can also be made with nuclear DNA, where the presence of rNMPs has been well documented to lead to both short TOP1-mediated deletions and other signs of genome instability [17, 20, 21]. The contrasting tolerance of rNMPs in mtDNA can be explained by at least three differing features between the mitochondrial and nuclear genomes. First, the considerable redundancy inferred by the multicopy nature of the mitochondrial genome makes it less vulnerable to strand breaks induced by rNMPs, as damage to one mtDNA molecule would still leave the cell with many intact copies. Should the reactivity of rNMPs result in a double-strand break, the damaged mtDNA molecule can be cleared by the exonuclease activity of the mitochondrial replicative DNA polymerase *γ* (Polγ) and by the MGME1 nuclease [47]. A second reason contributing to the rNMP tolerance in the mtDNA is the reverse transcriptase activity of Pol*γ* that allows it to read through a template-strand rNMP far more efficiently than nuclear replicative polymerases do, resulting in fewer termination events [46, 48–51]. The third contributing factor to the tolerance of rNMPs in the mtDNA is the lower activity of hTOP1MT at incorporated rNMPs uncovered in this study—thus, TOP1-mediated rNMP repair is far less likely to generate adverse outcomes like deletions in the mtDNA than in the nuclear DNA. Interestingly, others have reported mtDNA loss following artificial targeting of hTOP1 to human mitochondria [43]. However, the observed depletion was independent of hTOP1 catalytic activity, and may therefore be ascribed to tighter DNA binding or other disruptive actions of hTOP1 rather than cleavage at mtDNA rNMPs.

Our study did not address the activity of hTOP1MT on rNMPs embedded in the mtDNA *in cellulo*, so the potential impact of other mitochondrial proteins and/or cellular conditions on its endoribonuclease activity remains unknown. Second, available data indicate that the sequence preference of hTOP1MT largely aligns with that of hTOP1 and scTOP1, suggesting that the AGAT motif used in this study represents a relevant sequence motif to compare the enzymes’ activity on [19, 52, 53]. However, a more thorough analysis across a range of cleavage motif sequences is warranted. Another as-of-yet unanswered question is which residue(s) of hTOP1 and hTOP1MT contribute to the observed differences in their endoribonuclease activity. Further work is ongoing to answer these intriguing questions related to mtDNA stability that must be maintained in order to avoid disease.

## Supplementary Material

gkaf475_Supplemental_File

## Data Availability

The data underlying this article are available in the article and in its online supplementary material.

## Supplementary data

Supplementary data is available at NAR online.
