# Supplementary material for: The low endoribonuclease activity and lack of rNMP preference of human mitochondrial topoisomerase 1 protect against ribonucleotide-dependent deletions
Source: Nucleic Acids Res. 2025 Jun 6;53(11):gkaf475. doi: 10.1093/nar/gkaf475 (PMC12143597; doi:10.1093/nar/gkaf475)
Supplement: gkaf475_Supplemental_File [file gkaf475_supplemental_file.pdf]

Supplementary material

The low endoribonuclease activity and lack of rNMP preference of human mitochondrial topoisomerase 1 protect against ribonucleotide-dependent deletions

Cyrielle P. J. Bader[1,*], Erika Miyazaki-Kasho[1,*], Josefin M. E. Forslund[1], Aiswarya Dash[1], Malgorzata Wessels[1] and Paulina H. Wanrooij[1,‡]

Supplementary tables

Table S1. Oligonucleotides used in this study

The TOP1 consensus motif is underlined; the identity and position of potential fluorescent labels is indicated in each respective figure.

Name	Sequence
A	5'-AGC GTT GAA GAT ATG TGG CAA AAC CTT TGT-3'
B	5'-AGC GTT GAA GA**rU** ATG TGG CAA AAC CTT TGT-3'
Rev. comp. AB	5'-ACA AAG GTT TTG CCA CAT ATC TTC AAC GCT-3'
C	5'-ACT CGT CAC GAG AGA GAT GCC ACG GTA TTT CAA A-3'
D	5'-ACT CGT CAC GAG AGA **rG**AT GCC ACG GTA TTT CAA A-3'
Rev. comp. CD	5'-TTT GAA ATA CCG TGG CAT CTC TCT CGT GAC GAG T-3'
E	5'-ACT CGT CAC GAG AGA **T**A TGC CAC GGT ATT TCA AA-3'
F	5'-ACT CGT CAC GAG AGA **rU**A TGC CAC GGT ATT TCA AA-3'
Rev. comp. EF	5'-TTT GAA ATA CCG TGG CAT ATC TCT CGT GAC GAG T-3'

Table S2. DNA binding affinities of TOP1 enzymes to substrates A-D. The mean ± the standard error of the mean of 3-5 experiments; p-values are from comparisons using two-way ANOVA.

K_d (nM)	**hTOP1MT**	**hTOP1**	**scTOP1**	**hTOP1MT *vs.* hTOP1**	**hTOP1MT *vs.* scTOP1**	**hTOP1 *vs.* scTOP1**
**Sub A**	8.3 ± 0.59	0.97 ± 0.06	4.6 ± 0.25	<0.0001	<0.0001	<0.0001
**Sub B**	6.8 ± 0.18	2.02 ± 0.13	5.5 ± 0.67	<0.0001	0.1738	<0.0001
**Sub C**	4.7 ± 0.83	1.8 ± 0.05	6.6 ± 0.26	0.0006	0.0215	<0.0001
**Sub D**	4.6 ± 0.37	1.2 ± 0.09	6.0 ± 0.41	<0.0001	0.0900	<0.0001

Supplementary figure legends

Supplementary Figure 1, related to Figures 2-4. The purity and relaxation activity of scTOP1. (A-B) Quantification of the 18-mer product from reactions without CPT on substrate A **(A)** and B **(B)** from Fig. 2b and d. **(C-D)** Quantification of the 16-mer product from reactions without CPT on substrate C **(C)** and D **(D)** from Fig. 3b and d. **(E-F)** Quantification of the 18-mer product from reactions without CPT on substrate C **(E)** and D **(F)** from Fig. 3b and d. **(G)** SDS-PAGE analysis of the wildtype and catalytically-dead variants of the *Saccharomyces cerevisiae* TOP1 (scTOP1). **(H)** Relaxation reactions containing 350 ng of pUC19 and increasing concentrations (0.1, 0.5, 2.5 and 12.5 nM) of wildtype (*left panel*) or catalytically-inactive (*right panel*) scTOP1 enzymes. **(I)** Quantification of the relaxation activity of the wildtype scTOP1 from Fig. S1h, along with the wildtype hTOP1MT and hTOP1 from Fig. 1c. The amount of relaxed product was quantified and expressed in percent of the total signal intensity in the lane. The average of three independent experiments is shown, and the error bars represent the standard error of the mean.

Supplementary Figure 2, related to Figure 4. Fluorescence anisotropy for determination of DNA binding affinity of hTOP1MT, hTOP1 and scTOP1. (A) The sequences of the 5′-FAM labelled top strands of the dsDNA substrates used for the fluorescence anisotropy measurements in Fig. S2b-c. **(B-C)** Fluorescence anisotropy curves for reactions containing 0.5 nM substrate A **(B)** or substrate B **(C)** along with increasing concentrations (0.5, 1, 2, 4, 6, 8, 12, 16, 20, 30, 40, 60 nM) of catalytically-dead TOP1 variants (hTOP1MT-Y559F, hTOP1-Y723F and scTOP1-Y272F). **(D)** The sequences of the 5′-FAM labelled top strands of the dsDNA substrates used for the fluorescence anisotropy measurements in Fig. S2e-f. **(E-F)** Quantification of the DNA binding affinity of the TOP1 enzymes performed as in Fig. S2b-c but with substrate C **(E)** or substrate D **(F)**. The average of three independent experiments is shown, and the error bars represent the standard error of the mean.

Supplementary Figure 3, related to Figure 5. No deletion formation on an all-DNA substrate, and scTOP1 cleaves at an rG upstream of the cleavage site. (A) Representative TOP1 cleavage assay containing 50 nM substrate E and increasing concentrations (0.15, 0.5, 5, 15, 50 and 150 nM) of wildtype hTOP1MT, hTOP1 and scTOP1 enzymes in the absence of CPT. **(B)** Representative TOP1 cleavage assay containing 50 nM substrate E (*left panel*) and 150 nM wildtype hTOP1MT, hTOP1 and scTOP1 enzymes in the presence of 10 µM CPT. *Right panel:* quantification of the 18-mer cleavage product. **(C)** Representative TOP1 cleavage assay containing 50 nM substrate F and increasing concentrations (0.15, 0.5, 5, 15, 50 and 150 nM) of wildtype hTOP1MT, hTOP1 and scTOP1 enzymes in the presence of 10 µM CPT. **(D)** Quantification of the 18-mer product from 2 independent experiments including the one in Fig. S3c. **(E-F)** Representative TOP1 cleavage assay on substrate C without an rNMP **(E)** or substrate D with an rG upstream of the cleavage site **(F)** and increasing concentrations (0.15, 0.5, 5, 15, 50 and 150 nM) of wildtype scTOP1 in the absence of CPT. **(G)** Quantification of the 18-mer cleavage product in the reactions in Fig. S3e-f. The amount of the 18-mer product was quantified and expressed in percent of the total signal intensity in the lane. If not otherwise indicated, the average of the three independent experiments is shown, and the error bars represent the standard error of the mean.

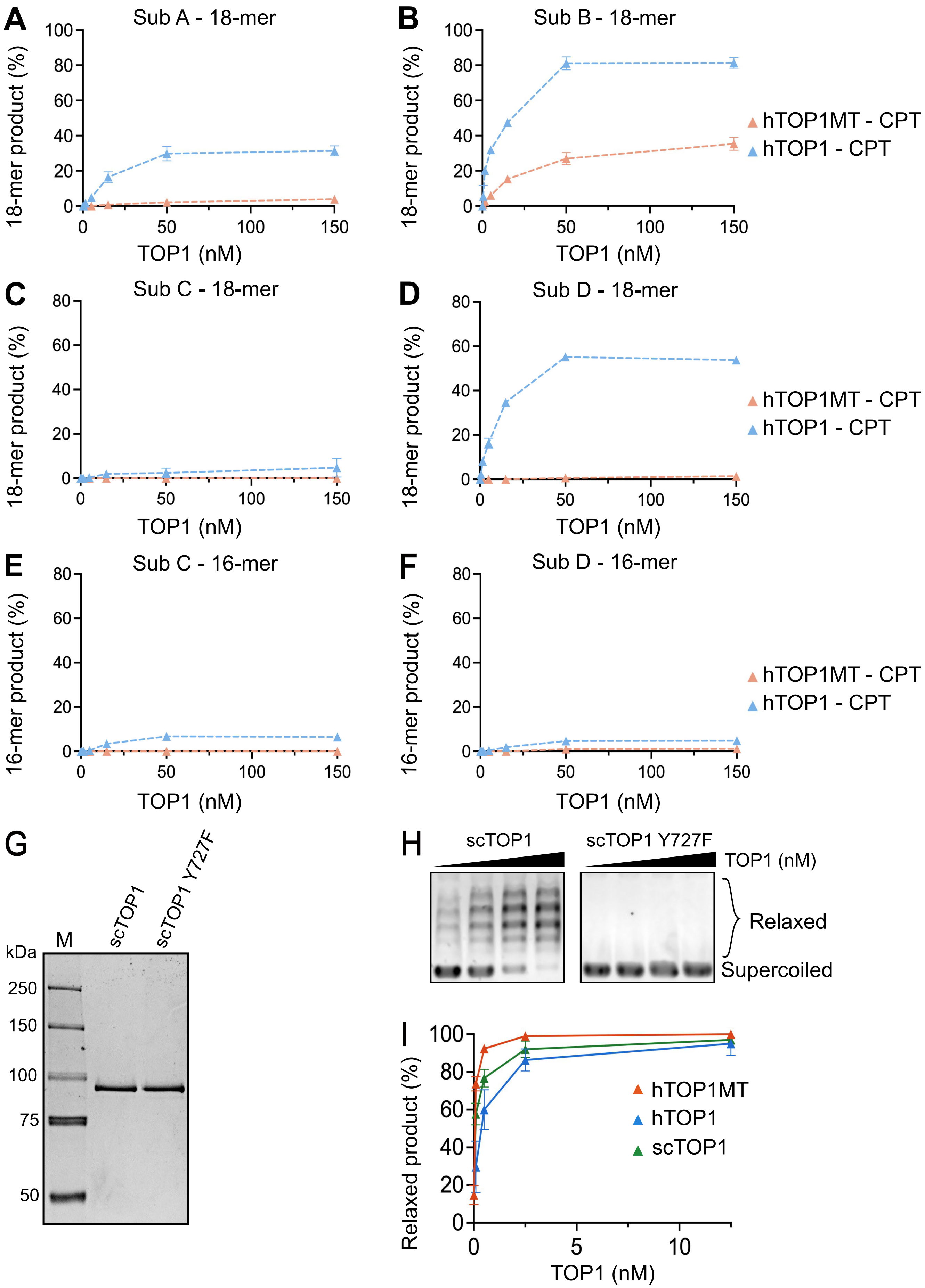

A
Sub A - 18-mer
18-mer product (%)
TOP1 (nM)
B
Sub B - 18-mer
hTOP1MT - CPT
hTOP1 - CPT
C
Sub C - 18-mer
D
Sub D - 18-mer
E
Sub C - 16-mer
16-mer product (%)
F
Sub D - 16-mer
G
M
scTOP1
scTOP1 Y727F
kDa
250
150
100
75
50
H
scTOP1
scTOP1 Y727F
TOP1 (nM)
Relaxed
Supercoiled
I
Relaxed product (%)
hTOP1MT
hTOP1
scTOP1

Fig. S1

A Sub A: 5' - FAM - AG CGT TGA **AGAT** ATG TGG CAA AAC CTT TGT - 3'

Sub B: 5' - FAM - AG CGT TGA **AGArU** ATG TGG CAA AAC CTT TGT - 3'

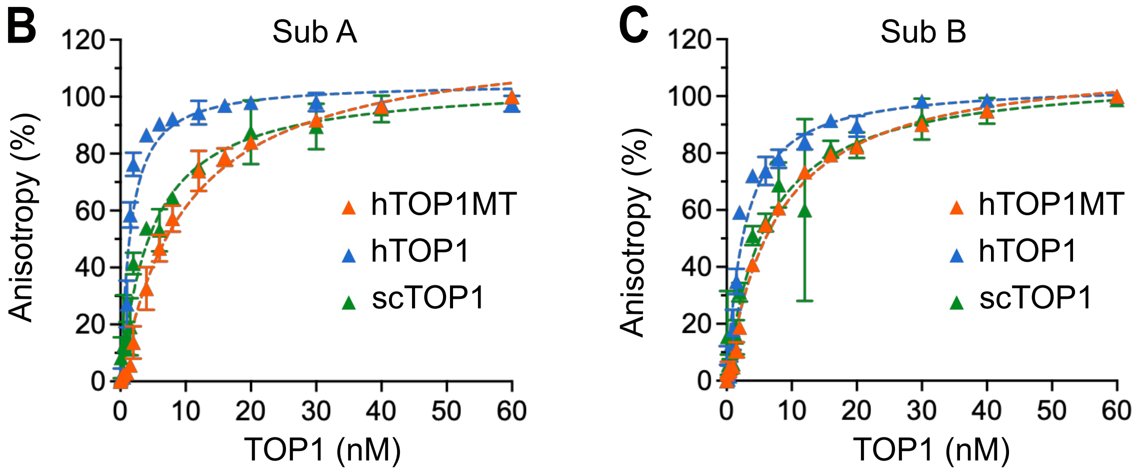

D Sub C: 5' - FAM - AC TCG TCA CGA GAG **AGAT** GCC ACG GTA TTT CAA A - 3'

Sub D: 5' - FAM - AC TCG TCA CGA GAG **ArGAT** GCC ACG GTA TTT CAA A - 3'

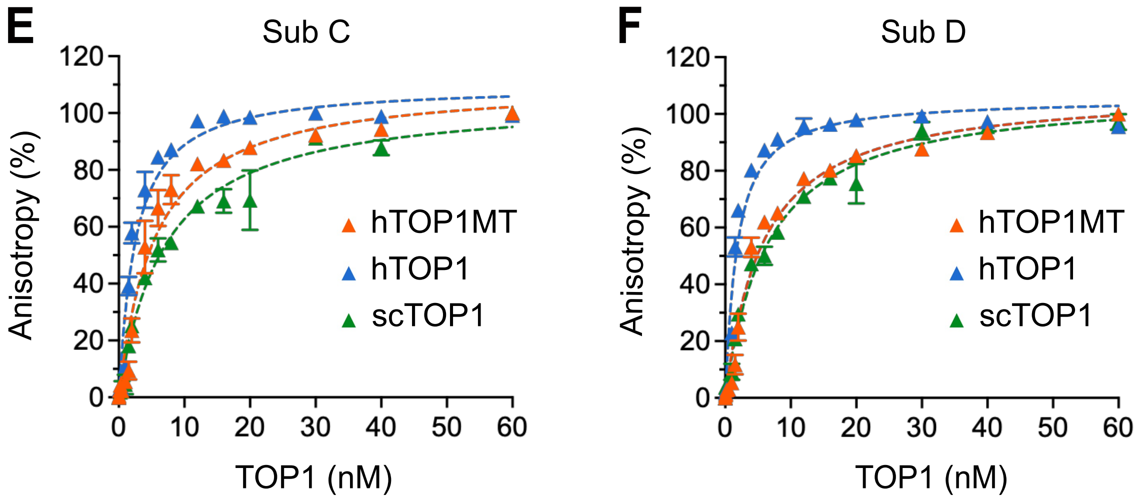

Fig. S2

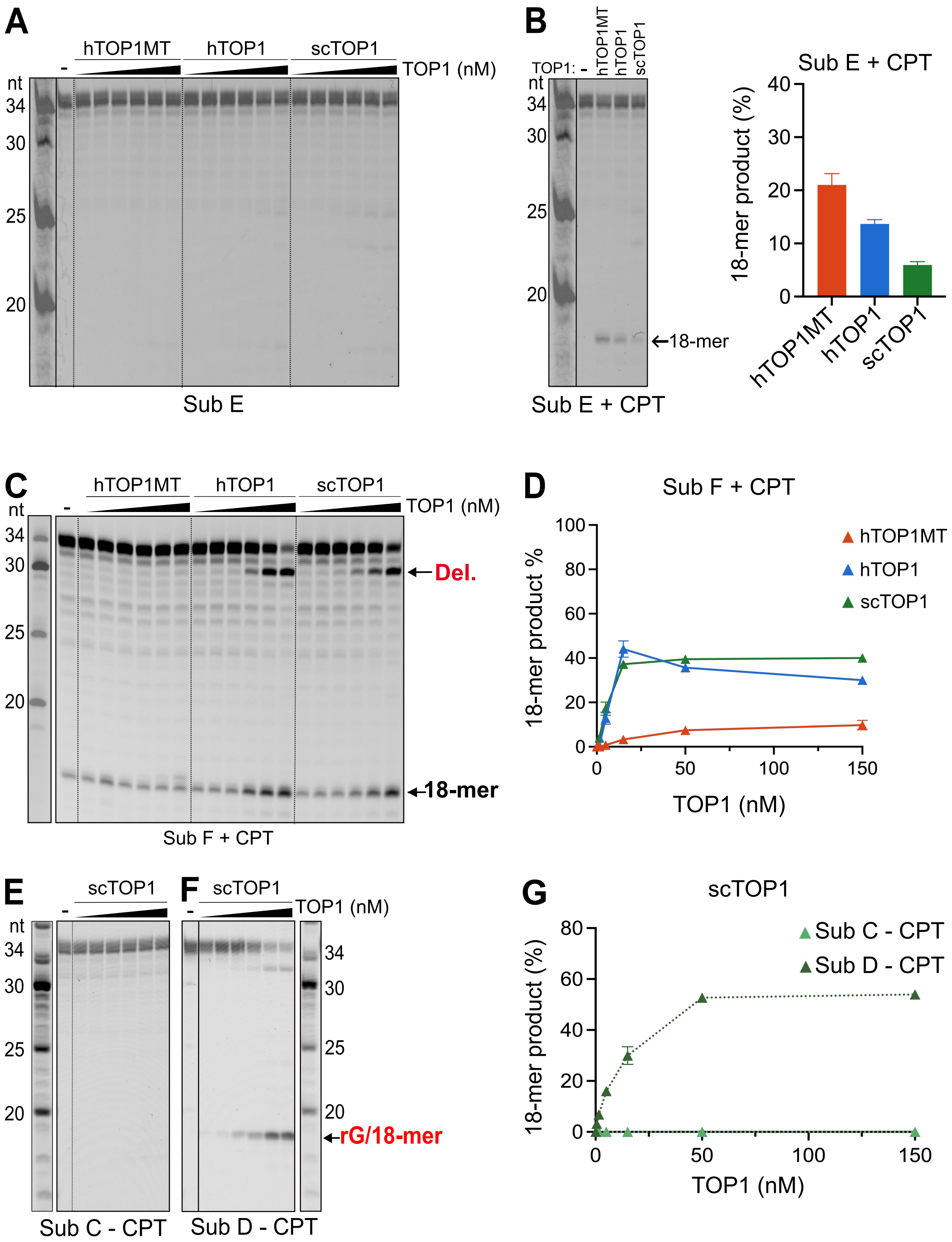
A
hTOP1MT
hTOP1
scTOP1
TOP1 (nM)
nt
34
30
25
20
Sub E
B
TOP1: - hTOP1MT hTOP1 scTOP1
18-mer
Sub E + CPT
Sub E + CPT
18-mer product (%)
hTOP1MT
hTOP1
scTOP1
C
Del.
18-mer
Sub F + CPT
D
Sub F + CPT
18-mer product %
TOP1 (nM)
E
F
scTOP1
rG/18-mer
Sub C - CPT
Sub D - CPT
G
scTOP1
Sub C - CPT
Sub D - CPT
18-mer product (%)
TOP1 (nM)

Fig. S3
